# Considerations for pediatric burn sedation and analgesia

**DOI:** 10.1186/s41038-017-0094-8

**Published:** 2017-10-16

**Authors:** Alice Fagin, Tina L. Palmieri

**Affiliations:** 1 Arkansas Children’s Hospital, 1 Children’s Way, Little Rock, AR 72202 USA; 2 Shriners Hospitals for Children Northern California and University of California Davis, 2425 Stockton Blvd, Suite 718, Sacramento, CA USA

**Keywords:** Pediatric, Sedation, Burns

## Abstract

Burn patients experience anxiety and pain in the course of their injury, treatment, and recovery. Hence, treatment of anxiety and pain is paramount after burn injury. Children, in particular, pose challenges in anxiety and pain management due to their unique physiologic, psychologic, and anatomic status. Burn injuries further complicate pain management and sedation as such injuries can have effects on medication response and elimination. Burn injuries further complicate pain management and sedation as such injuries can have effects on medication response and elimination. The purpose of this review is to describe the challenges associated with management of anxiety, pain, and sedation in burned children and to describe the different options for treatment of anxiety and pain in burned children.

## Background

Appropriate sedation and use of sedative medications is extremely important in pediatric burn patient due to the pain, anxiety, fear of strangers, separation from parents, and loss of control that can accompany burn injury [1–3]. Additional anxiety related to hospitalization, social situation, and long-term body image compound the problem [4]. Frequent procedures and interventions, in which children cannot cooperate with or understand the need for, make the pediatric burn patient rife for significant anxiety and distress both at the time of and after injury [5]. Left untreated, anxiety can intensify into a pathway of fear, sleeplessness, depression, and helplessness that may render patients psychologically incapable of coping with their illness or treatment at the time of and after burn injury [4]. Early burn research contributed to the original classification of posttraumatic stress disorder in DSM-III; post-traumatic stress disorder criteria are met in 30% of severely burned children 6 months after injury [1, 6, 7].

The purpose of this review is to define sedation and analgesia, define how burn injury impacts analgesic requirements, and describe the current pharmacologic and nonpharmacologic methods of treating pain and anxiety in children with burn injuries.

## Review

### Definition of terms

Understanding the differences between pain, distress, and fear is essential to establishing appropriate treatment algorithms for optimal pediatric burn sedation.

Distress is defined as “an organism’s response to aversive internal and external stimuli” [8]. Anxiety is a response based on worry or apprehension, which may be linked to a perceived or real threat. Agitation is identified as a more intense level of nervous anxiety [9]. Fear differs in that it is an unpleasant emotion due to the belief that someone/thing is dangerous, may cause pain, or is a threat.

Fear and anxiety may be difficult for medical providers to distinguish from pain, particularly in younger children [7]. Distress and pain are difficult to discriminate, as these experiences may occur simultaneously, influence each other, and present with comparable responses [8]. Burn pain, specifically, is one of the most severe forms of acute pain [10]. Uncontrolled moderate to severe pain can impede proper wound care, physical therapy, and lengthen hospitalization [4, 11]. A primary medical task of the burn unit is the relief of pain and anxiety, each of which can exacerbate the other [12].

Sedation is defined as a calm tranquil state that allays anxiety and excitement [13]. Sedation is further described as a medical procedure involving administration of sedative drugs, generally to facilitate a medical procedure, such as endoscopy, vasectomy, or minor surgery with local anesthesia [14]. As such, sedation can be used to allay distress and/or fear. The first known use of sedation was in 1543 [15].

### Non-pharmacologic interventions for distress (or Anxiety), pain, and fear

Initially, anxiety should be allayed with attempts to normalize the child’s surroundings with non-pharmacologic interventions. Communication, continuous reorientation, reassurance, and the presence of relatives at the bedside can allay anxiety, while environmental factors such as noise reduction, utilization of adequately lighting to promote an adequate sleep awake cycle, promoting time to rest and sleep to maintain a circadian orientation, restricting procedures to daytime, keeping the patient in a comfortable position using cushions, and attention to fluids and feeding may improve comfort [16–18]. Non-pharmacologic interventions to reduce stress, such as live or recorded music, have primarily been studied in adults, but make sense for children as well [16]. Use of earplugs, eye masks, noise reduction, and darkness for sleep promotion is uncommon in critical care settings; however, efforts to normalize the patient’s routine may allow the child to feel more secure and less anxious [5].

The normal young child spends a majority of his or her time in play. When children are confined to their ICU bed, both their schedule and their sense of self and normalcy are compromised. The play specialist has an important role in the pain, fear, and distress management in the Pediatric and Burn ICU, assessing children and introducing individualized distraction therapy [17].

Beyond scheduling, the hospital environment is significantly different from a child’s daily norm. Light and noise, for example, can be disturbing. Allowing children to wear ear plugs, asking staff to speak softly, and preventing ongoing alarm sounds can be helpful [16]. Environmental noise in critical care units ranges from 60 to 84 dB, with the World Health Organization defining 55 dB as serious annoyance [17]. For children, in an environment that is foreign, it is even more important to provide calm soothing sounds to minimize disturbances or provoke nighttime fears [16].

### Pharmacologic interventions for sedation

Non-pharmacologic interventions continue to have importance in the care of the burned child, but frequently are inadequate to fully treat pain and anxiety. Analgesia and sedation are essential elements in patient care in the ICU to control pain, anxiety, and agitation; prevent the loss of devices or accidental extubation; and improve the synchrony of the patient with mechanical ventilation [16, 18, 19]. The ideal sedative agent should have rapid onset, short duration of action, minimal active metabolites, few side effects, predictable pharmacokinetics (PK), and cost-effectiveness [1, 20]. Additional issues to consider are hemodynamic changes, patient and family satisfaction, ease of maintaining desired level of sedation, recovery time, and risk of adverse events. To achieve the best possible outcome, interdisciplinary collaboration of nurses, physicians, and hospital pharmacists/clinical pharmacologists is warranted [21].

Sedation is a wide-ranging topic with multiple subcategories. There are two main types of sedation in burn patients: procedural sedation and ongoing sedation, particularly in the intubated patient.

### Procedural sedation

Procedural sedation is defined as using amnestic, anxiolytic, or analgesic agents to prevent the child from remembering or feeling painful procedures [22]. The number of noninvasive and minimally invasive procedures performed outside of the operating room has grown exponentially over the last several decades [11, 23]. Although procedures are an integral part of the care of burn patients, they can also be disruptive. Regular burn wound care procedures can cause extreme pain and fear, which can result in posttraumatic stress disorder, psychological sequelae, or chronic pain [11, 24–27]. Percent of total body surface burned is associated with increased procedural anxiety [4].

Once sedation is determined to be necessary, the provider needs to determine the depth of sedation that will be required (Table 1). Minimal sedation (anxiolysis) is a drug-induced state during which patients respond normally to verbal commands. Although cognitive function and physical coordination may be impaired, airway reflexes and ventilatory and cardiovascular functions are unaffected [28]. This level of sedation is ideal for non-pharmacologic modalities. Moderate sedation (conscious sedation) is a drug-induced depression of consciousness during which patients respond purposefully to verbal commands, either alone or accompanied by light tactile stimulation. No interventions are required to maintain a patent airway, spontaneous ventilation is adequate, and cardiovascular function is usually maintained [28]. Patients should be monitored continuously with noninterruptive technologies such as continuous pulse oximetry [29]. Deep sedation is a drug-induced depression of consciousness during which patients cannot be easily aroused but respond purposefully following repeated or painful stimulation. Although cardiac function is maintained, the ability to independently maintain respiratory function may be impaired; hence, the patient may require assistance in maintaining a patent airway, and spontaneous ventilation may be inadequate [28]. Deep sedation for children, with its higher risks of complication, requires use of the appropriate drug that is prescribed and administered by qualified personnel under strict guidelines to avoid potential complications [30]. For wound care, any sedation above the minimal level requires qualified personnel prepared to manage an airway, given that a greater depth of anesthesia may occur than what was anticipated/planned.

**Table 1:** Sedation levels (DDD)

	Minimal sedation	Moderate sedation	Deep sedation	General anesthesia
Responsiveness	Normal response to verbal stimulation	Purposeful response to verbal or tactile stimulation	Purposeful response following repeated or painful stimulation	Unarousable even with painful stimulus
Airway	Unaffected	No intervention required	Intervention may be required	Intervention often required
Spontaneous Ventilation	Unaffected	Adequate	May be inadequate	Frequently inadequate
Cardiovascular function	Unaffected	Usually maintained	Usually maintained	May be impaired

Pediatric sedation and analgesia practices differ from adults due to characteristics inherent to children, including small size and cognitive immaturity [31]. Serious associated risks are associated with pediatric sedation including hypoventilation, apnea, airway obstruction, laryngospasm, and cardiopulmonary impairment [32]. The risks increase as the level of sedation deepens. Further, adverse outcomes may be increased when two or more sedating medications are administered [23]. And since every child is different, the risks will vary. In particular, children with developmental disabilities have a threefold increased incidence of desaturation compared with children without developmental disabilities [23]. Challenges for sedation in burned children, not presented in other population, include donor site pain and complex wound care, inhalation injury, presence of burn hypermetabolism, and pain associated with burn wounds [31]. Burn injury markedly alters the PK and pharmacodynamics (PD) of many drugs [33].

The stress of inpatient treatment, specifically a burn dressing change, has been compared with “inescapable shock” or “learned helplessness” for pediatric burn patients [12]. The degree of dissociative symptoms measured shortly after the burn is a direct predictor of posttraumatic stress disorder symptoms [6]. Early usage of appropriate sedation for burn wound care allows for early aggressive wound debridement, virtually eliminating the need for operating room debridement, and may eliminate patient discomfort and fear often associated with subsequent debridements [22].

### Ongoing sedation in the ventilated patient

Perhaps one of the most challenging components of pediatric critical care is sedation of mechanically ventilated children. The goal of sedation for both intubated and nonintubated patients is to attain a calm but responsive state that protects the young patient from self-harm. An ideal level of sedation in ventilated patients, however, is described as a state in which the patient is sleepy, responding to environmental stimuli, without risks and excessive movement [18, 21]. In an intubated patient, this means that a child is conscious, breathes in synergy with the ventilator, and is tolerant or compliant to other therapeutic procedures [21]. An arousal target of light sedation in this setting is most likely to improve patient outcome from critical illness [34]. Administration of sedatives to critically ill patients may be complicated by unpredictable PK and PD due to internal factors (impaired organ function, drug interactions, altered protein binding, and fluctuating volumes of distribution) [16, 25]. External factors that can change PK and PD of sedative drugs include renal replacement therapy, ECMO, and hypothermia [16]. The age distribution of children admitted to pediatric intensive care units with associated differences in metabolism of many drugs due to variable enzyme kinetics complicates optimal dosing [35].

Appropriate sedative and analgesic therapy of intubated, critically injured, pediatric burn patients is challenging due to the severity of the child’s illness, burn-associated alterations in drug metabolism, and rapid development of tolerance to the most commonly used sedative agents [24]. Burn injury also alters the metabolic clearance of many sedatives and analgesics [24]. The stress caused by the burn injury itself, together with the co-administration of opiates and sedatives, can induce tolerance and even opiate-induced hyperalgesia [24]. Thus, in patients who need protracted mechanical ventilation, the “normal” doses of benzodiazepine and opiate medications often become inadequate, resulting in dose escalation or addition of other drugs, to achieve effectively the desired effect.

The inability to communicate is compounded by the child’s cognitive limitations, making sedation particularly challenging [7]. Over 90% of infants and children supported on mechanical ventilation receive some form of sedative therapy [8]. Appropriate use of sedatives, in these difficult-to-treat patients, reduces anxiety, optimizes patients’ comfort, and improves outcomes [20]. Contrary to procedural sedation, which has a fixed duration and specific purpose, ongoing sedation has indeterminate duration and sedation depth. Also, unlike procedural sedation, continuous sedation infusion is but one part of a multilayered and complex care plan [36].

Lack of consensus guidelines for sedation and analgesia delivery to pediatric intensive care unit patients result in practice variation, and regional attitudes can influence the practices and management of sedation [16, 32, 37]. To achieve optimal sedation in critically ill patients, the use of sedation guidelines, protocols, and algorithms has been advocated by various societies as a means of improving practice and standardizing care [19]. The level of sedation should be regularly assessed and documented using a validated scoring system, each patient’s desired level of sedation should be identified and regularly reassessed, and doses of sedative agents should be titrated to produce the desired level of sedation [17]. A system for determining sedation efficacy is important because sedation is a continuum and individual patient responses are unpredictable [38]. Despite widespread recommendation for the use of sedation guidelines, protocols, and algorithms in critically ill children, there is a paucity of high-quality evidence to guide this practice. More robust studies are urgently needed [19].

Inadequate sedation, whether insufficient or excessive, has common side effects, such as an increase in the duration of mechanical ventilation, hospital-acquired infections (in particular ventilator associated pneumonia), hemodynamic instability, unplanned extubation or failure of extubation, development of withdrawal syndromes, and long-term adverse neuropsychological outcomes [8, 16, 37]. Excess sedative medications increase morbidity and mortality [18]. Profound sedation in the first 24 h increases mechanical ventilation duration by 12 h, in-hospital mortality by 10%, and 180 day mortality by 8% [34]. Insufficient or excessive sedation is likely to add to the personal and financial burden of intensive care [13].

In one study, optimal sedation was achieved in 57.6% of sedation assessments, undersedation in 10.6% and oversedation in 31.8% [39]. After implementation of protocols for systematic management of sedation, analgesia, and delirium, the mean ICU length of stay and duration of mechanical ventilation is shorter (5.43 vs 6.39 and 5.95 vs 7.27) and the proportion of patients with appropriate sedation increases from 57 to 66.6% [40]. Patients who were managed using sedation protocols are 23% more likely to be taken off all sedation compared to physician-directed approaches [41], and the median duration of sedation is reduced in sedation guidelines [42]. All members of the complex critical care team, doctors, nurses, therapists, and techs, need to have a common understanding of the goals of sedation.

### Medications

The appropriate techniques for sedation/analgesia are dependent on the experience and preference of the individual practitioner, requirements, or constraints imposed by the patient or procedure, and the likelihood of producing a deeper level of sedation than anticipated [43]. The choice of sedative agent depends on the efficacy and safety profile of the agent as well as its relative safety and efficacy compared to other medications [44]. For burn patients specifically, one must not only consider the age of the patient but also the location of the burn, burn depth, burn extent (percent), and time of debridement [22]. Comparison of common usage dosages and adverse effects appears in Tables 2 and 3.

**Table 2:** Commonly used dosages for common sedative agents

Medication	Common dosages (continuous)	Common dosages (procedural)
Midazolam	0.06–0.12 mg/kg/h	0.25–0.5 mg/kg by mouth 30 min prior
Dexmedetomidine	0.2–1.5 mcg/kg/h	Loading 1 mcg/kg IV over 10 min followed by maintenance 0.6 mcg/kg/h
Propofol		2.5–3.5 mg/kg IV over 20–30 s followed by 125–300 mcg/kg/min
Ketamine	2 mcg/kg/min for opioid sparing	1–4.5 mg/kg IV or IM, additional dosages 0.5–1 mg/kg as needed
Haloperidol	0.5 mg/day by mouth in 2–3 divided doses, may increase every 5–7 days until desired response

**Table 3:** Common adverse effects for common sedative agents

Medication	Common adverse effects
Midazolam	Hypotension, respiratory depression, oversedation, significant risk of tolerance
Dexmedetomidine	Bradycardia, hypotension, nausea/vomiting, fever, hypoxia, anemia
Propofol	Propofol infusion syndrome (severe metabolic acidosis, hyperkalemia, hyperlipidemia, rhabdomyolysis and organ failure)
Ketamine	Airway obstruction, laryngospasm, respiratory depression, tachycardia, hypotension, emergence delirium, hypersalivation
Haloperidol	Acute dystonic reactions, parkinsonian reactions, body temperature dysregulation, akathisia

The lowest dose of a drug with the highest therapeutic index for the procedure should be administered [32]. Selection of the fewest number of drugs and matching drug characteristics to the type and goal of the procedure are essential to safe practice [32], because the potential for adverse outcome may be increased when three or more sedating medications are administered [32]. Common medications used for sedation in the ICU include opioids, benzodiazepines, ketamine, and alpha-agonists [7]. A survey of burn centers found that the most common sedative agents used by pediatric burn clinicians were midazolam, ketamine, and diphenhydramine [31].

### Midazolam

Midazolam is the most commonly used agent for continuous sedation [45], as well as for procedural sedation [44]. It is frequently considered first-line treatment for reducing fear and anxiety in burn patients [46]. Benzodiazepines increase the affinity of the inhibitory neurotransmitter gamma-amino butyric acid (GABA) for cell surface receptors located on post-synaptic neurons in the spinal cord dorsal horn and brain stem, leading to increased frequency of chloride channel opening and prolongation its open state [47, 48]. Midazolam is characterized by rapid onset, high potency, and short duration [5, 11, 16, 17, 22]. Due to its water-based acidic preparation, at plasma pH, it converts into an un-ionized form that crosses the blood brain barrier rapidly [17]. Midazolam has a fast onset of action of 1–5 min following IV administration with an elimination half-life of approximately 1–3 h [16, 49]. The effects last for 30–120 min after a single infusion and up to 48 h after 1 week of continuous infusion [16, 17].

Midazolam produces anterograde and retrograde amnesia (without impairing the ability to retrieve previously learned information), muscle relaxation, anxiolysis, and sedation, but lacks analgesic properties [1, 5, 16, 17, 46, 49]. Thus, it may not be a sufficient sedative on its own and is frequently paired with narcotics [43]. Synergy with opiates to relieve the distress associated with large burns makes careful benzodiazepine use very appropriate in these patients [12]. Physiologic effects at therapeutic doses include a slight reduction in heart rate, systemic vascular resistance, and a small reduction in tidal volume with a compensatory increased respiratory rate. Potential complications thus include hypotension, respiratory depression, and over sedation [2, 49].

Midazolam requires additional caution in dose calculations with children to avoid unwanted side effects, especially when used in combination with narcotics or other CNS depressants [11]. The pharmacokinetics and pharmacodynamics have been shown to change with age, leading to significant inter-individual variability [5]. Care must be taken in dosing patients with hepatic or renal failure as midazolam accumulates in these patients [16]. Critical illness alone variably reduces midazolam clearance independently of serum creatinine levels and could increase sedation depth [16]. Additionally, midazolam has active metabolites, notably α-hydroxymidazolam and gluycuronidated α-hydroxymidazolam; the conjugated metabolite accumulates to sedative concentrations with prolonged midazolam administration [49].

Long-term use of midazolam results in downregulation of receptors with decreased function as determined by chloride uptake [48]. Often, children receiving the drug for prolonged periods of time develop tolerance, requiring higher and higher doses for the same effect [50], which increases the risk of unwanted effects. Sedation with benzodiazepines is associated with longer ICU length of stay than sedation with non-benzodiazepines as well as an increased risk of delirium [5, 34].

The advantage of benzodiazepines is the availability of a reversal agent, flumazenil, which is a competitive inhibitor at the benzodiazepine receptor site [22, 49]. Flumazenil also reverses the active metabolites of the midazolam [49]. Flumazenil is contraindicated in patients who have received chronic benzodiazepine therapy as it may precipitate acute withdrawal seizures in these patients.

### Dexmedetomidine

Dexmedetomidine is a newer drug which is less deliriogenic compared with benzodiazepines [20]. It is a highly selective alpha-2 adrenoreceptor agonist used for sedation due to its anxiolytic and analgesic properties without respiratory compromise [5, 18, 20, 24, 40, 51–53]. Dexmedetomidine has an alpha-2/alpha-1 receptor affinity eight times that of the closely related drug, clonidine [52]. Dexmedetomidine works primarily by activating receptors in the locus ceruleus of the brain stem [2, 54], which reduces sympathetic outflow [16].

The sedative effects of dexmedetomidine are well documented when given as an intravenous bolus, continuous infusion, or intramuscular injection. The drug is tasteless, odorless, and painless when administered intranasally [54]. Oral routes of dexmedetomidine administration have a bioavailability of 17%, the onset of effects is variable, and use is limited by cost for quantity of dose [54]. The drug is metabolized in the liver then secreted primarily in the urine without active or toxic metabolites [2]. The elimination half-life is approximately 2 h and duration of action is 4 h (52). Terminal half-life of dexmedetomidine in the plasma is approximately 180 min, with a context sensitive time of approximately 30 to 45 min until awakening when given intravenously [54]. Recommended adult dosing is 0.2 to 0.7 μg/kg/h, but needs have been observed in pediatric burn patients to be much higher, up to 2.5 μg/kg/h [24]. Dexmedetomidine exhibits linear kinetics when infused in the recommended dose rate [52].

Dexmedetomidine produces “arousable sedation” whereby patients experience clinically effective sedation but are easily arousable [2]. It provides sedation resembling natural sleep [5, 51, 52]. The desired effects of anxiolysis, reduced delirium, and anti-shivering properties are achieved without respiratory depression [51]. At higher doses, it also prevents recall and memory [2]. Use of dexmedetomidine with its mild-to-moderate analgesic properties allows for opioid-sparing effects in adults [16, 24, 52]. There is potential application to decrease development of perioperative opioid-induced hyperalgesia that may develop in the critical care or burn pain setting where significant opioid use is often the norm for sedation and analgesia [46]. Overall, dexmedetomidine is very effective with lower levels of respiratory depression and fewer side effects [52].

Adverse reactions are primarily cardiogenic with bradycardia and sinus arrest associated with rapid intravenous administration [2]. Hypotension and bradycardia are particularly common with bolus dosing [20]. Additional possible side effects include hypertension, nausea, vomiting, fever, hypoxia, tachycardia, and anemia [52]. Dexmedetomidine overall seems to be well tolerated, and the cardiovascular side effects are well manageable [16].

Burn patients have persistent catecholamine surge extending several weeks after injury with circulating high levels of epinephrine, norepinephrine, and dopamine. The catecholamine responses to burn injury are compounded by increased renin-angiotensin activity, particularly in burned children. These endogenous vasoactive hormones result in hypertension or maintenance of normotension even during relative hypovolemia after burn injury. The use of any sedative drug in burned patients, who have high sympathetic tone, may require high fluid volumes to maintain normotension whether it be dexmedetomidine, midazolam, or morphine [24]. In a study of 42 pediatric burn patients, comparing dexmedetomidine to midazolam, hypotensive episodes were more common in the midazolam group (mean 29.7 vs 15.8) with midazolam having twice the number of incidents per day (1.5 vs 0.7). The single episode of bradycardia was asymptomatic and resolved with weaning of the dexmedetomidine infusion [2]. To minimize the risk of cardiovascular adverse effects, it may be prudent to avoid bolus dosing of Dexmedetomidine in the severely burned pediatric patient on the ventilator [24].

Overall, studies of dexmedetomidine in the burn patient have demonstrated beneficial effects of the drug in comparison to other modalities. In pediatric burn patients, dexmedetomidine achieved sedation more frequently in the ideal range with RASS of zero compared to midazolam (mean −0.9 vs −1.33) and more appropriate Riker scores than other sedatives [2, 20].

A double-blind multicenter trial randomized 375 mechanically ventilated adult ICU patients to receive dexmedetomidine or midazolam infusions. While the percent of time patients which were maintained in the target sedation range did not differ (77% vs 75%), patients treated with dexmedetomidine experienced a lower frequency and shorter duration of delirium, fewer infections, a lower rate of tachycardia and hypertension requiring treatment, and a shorter time to extubation [55].

An advantage of dexmedetomidine is that with preservation of respiratory drive, patients are able to be weaned from the ventilator and extubated while on dexmedetomidine drip [2, 52], which may decrease overall mechanical ventilator days [16]. With all the recent interest and research related to dexmedetomidine, it is becoming more and more common in the ICU. Dexmedetomidine has become a frequent choice for second- or third-line therapy and use is increasing [3]. The most commonly stated reason for not using dexmedetomidine is its cost, but the decrease in length of stay and dose required may start to mitigate the difference in cost.

### Propofol

Propofol is a short-acting, lipophilic intravenous general anesthetic that causes global CNS depression, presumably through agonism of GABA_A_ receptors and reduced glutamatergic activity through *N*-methyl-d-aspartate (NMDA) receptor blockade [36]. It is very rapid acting and versatile with a rapid clearance and smooth recovery [16, 31, 26]. Propofol may decrease arterial pressure due to a decreased systemic vascular resistance and cardiac contractility [26]. A main advantage of propofol is that recovery time and total sedation time are shorter than other treatment modalities [43]. Although propofol has gained popularity for short-term sedation, clinical experience is limited in extended treatment, especially in children [48].

Propofol has never been licensed for the provision of sedation in critically ill children and should not be used to provide continuous sedation in critically ill children [17]. A black box warning was issued in 2001 due to pediatric patient morbidity [31]. Long-term use in children is contraindicated as it may lead to propofol-infusion syndrome, which has a higher incidence in children than adults [16, 18]. Propofol infusion syndrome is a metabolic disorder with severe metabolic acidosis, hyperkalemia, hyperlipidemia, rhabdomyolysis, and organ failure, associated with an increased risk of mortality [16, 17]. Risk factors are doses >4 mg/kg/h with duration of >48 h, but short-term high doses can be problematic. Other risk factors include young age, critical illness, high fat and low carbohydrate intake, inborn errors of mitochondrial fatty, acid oxidation and concomitant catecholamine infusion or steroid therapy [16].

Propofol should be administered only by persons trained in the administration of general anesthesia and not involved in the conduct of the surgical/diagnostic procedure [35].

Despite these concerns, propofol continues to be used with increasing frequency in ICU procedures. In a 1997 survey, 22% of clinicians were using propofol to sedate pediatric patients but only for short periods of time [45]. In a subsequent 2015 survey of Canadian Pediatric ICUs (PICUs), propofol was used as a continuous infusion by at least 60% of respondents [3].

### Ketamine

Ketamine is an NMDA receptor antagonist that dissociates the cortex from the limbic system producing analgesia, sedation, and amnesia [1, 16, 30, 44]. Ketamine produces dose-related decreases in level of consciousness, culminating in general anesthesia [43]. Target serum concentration of 1 mg/L provides moderate sedation and concentration of 1.5 mg/L provides deep sedation [16]. Ketamine has consistently been found to be one of the most effective and safe medications for procedural sedation [44].

The major advantage of ketamine is that it usually preserves airway patency and respiratory function, without significant oxygen desaturation or clinically significant emergency reactions [10, 16, 29, 27, 46]. Hypotension also rarely occurs with administration, and ketamine has a wide margin of safety [12, 29].

Although it may be associated with less cardiorespiratory depression than other sedatives, airway obstruction, laryngospasm, and pulmonary aspiration may still occur with ketamine [43]. Additional potential side effects of ketamine include respiratory depression, tachycardia, hypertension, emergence delirium, and hypersalivation [16, 30, 22]. Additionally, there is a risk of increased intracranial pressure because of intracranial vasodilation [16]. The most frequently mentioned adverse effect of ketamine is emergence reactions or hallucinations [13]. These psychomimetric side effects are reduced in children [46]. Tolerance to the anesthetic effects of ketamine has been reported, and several studies demonstrate an increased dose requirement and/or decreased sleep time with the same dose of ketamine after repeated exposure to the medication [36, 48, 56].

Ketamine blocks NMDA receptor and may prevent opioid tolerance; therefore, ketamine may be an adjunct to sedatives and opioid analgesics as ketamine decreases opioid consumption by 30% in the postoperative surgical setting [16, 46]. Ketamine modulates opioid-induced hyperalgesia by modifying pronociceptive systems and affecting antinocioceptive systems, due to NMDA antagonism [46].

### Haloperidol

Haloperidol is a high-potency neuroleptic of the butyrophenone class that binds post-synaptic dopamine (D2) receptors in the mesolimbic pathway leading to restoration of hippocampal function [57, 58]. The major indication for the use of haloperidol is marked agitation and restlessness. Less common indication is use in delirium with marked disorientation, hallucinations, delirium, and delusions [57, 58]. Haloperidol has been used to manage critically ill pediatric burn patients when standard pain and anxiety treatment protocol prove insufficient. Haloperidol is less sedating compared with other antipsychotics and has minimal effects on blood pressure, heart rate, renal function, and respiration function [58].

Side effects of haloperidol include extrapyramidal symptoms such as acute dystonic reactions, parkinsonian reactions, body temperature dysregulation, and akathisia. Other less common adverse symptoms are seizures and the potentially fatal neuroleptic malignant syndrome [57]. Extrapyramidal symptoms often can be relieved by reduction in the dose of haloperidol. If symptoms persist, an anticholinergic agent or antihistaminic agent is added [57]. Approximately 90% of adverse events occur within 4 days of starting treatment with high potency neuroleptics and occur most often in children and young adults, especially boys [57].

Haloperidol can be administered by enteral, intramuscular, and intravenous route [57]. Enteral has 1st pass metabolism in the liver [57]. The half-life for serum levels varies between 12 and 22 h regardless of the route of administration, although the average is approximately 16 h [57].

Haloperidol is effective in children with positive outcomes in the treatment of psychotic symptoms [57]. Approximately 23% of children treated with haloperidol experienced adverse effects. Thus, haloperidol use should be closely monitored [57].

### Procedural sedation and combination sedation considerations

Combining a sedative with an opioid provides effective moderate sedation, particularly for procedures; however, it is unclear if the combination of a sedative and opioid are more effective than a sedative or an opioid alone in providing adequate moderate sedation [43]. Combinations of sedatives and opioids may increase the likelihood of adverse outcomes, including ventilator depression and hypoxemia [43]. Fixed combinations of sedative and analgesic agents may not allow the individual components of sedation/analgesia to be appropriately titrated to meet the individual patient and procedure requirements while reducing the associated risks [43].

Because of the synergistic effect of opioids and benzodiazepines, lower doses are typically adequate when used together [49]. Midazolam doses may be reduced by as much as 30–50% when combined with opioid [49]. Future studies need to explore the mechanisms of interaction between different benzodiazepines and opioids to identify effective drug combinations with effective sedation and analgesia but fewer side effects [47].

Combination pharmacologic regimens with analgesic, amnesic, and anxiolytic effects offer a broader range of physical and psychological pain management than using a single pharmacologic agent for management of pain during burn wound care [11]. In pediatric burn patients, the combination of midazolam and ketamine produces better premedication than either drug administered alone [1]. More children, age 1–5, who received midazolam and ketamine orally, achieved an adequate level of sedation than in the group that received midazolam, acetaminophen, and codeine [1]. While adverse reactions were significantly higher in the midazolam/ketamine group, they were of minor clinical significance and did not compromise patient stability [1]. Ketamine-midazolam therapy is associated with fewer adverse effects than other common parenteral drug combinations [44].

In burn patients, oral clonidine produces effective analgesia and sedation with intravenous ketamine by reducing sympathetic outflow generated by ketamine [59].

Using a similar mechanism, dexmedetomidine attenuates the cardiostimulatory, psychological, and CNS effects of ketamine [27, 60]. The combination of ketamine/dexmedetomidine has potent antinociceptive activity and may result in a decrease in the total drug dose. During a study of pediatric burn dressing changes, ketamine/dexmedetomidine showed significant increase in systolic blood pressure values after induction. Ketamine/dexmedetomidine can be considered as an excellent alternative for pediatric wound dressing changes [27].

Greater than 90% of infants and children supported on mechanical ventilation receive psychoactive medications, most commonly combinations of opioids and benzodiazepines [5, 37, 58]. Continued research is required to determine the best combination for ongoing pediatric sedation for mechanical ventilation.

### Complications of long-term sedation

While the need for sedation is real and significant, sedation has risks. While we have already covered the unintended consequences of short-term procedural sedation related to over- or under-sedation, the most noted complications of long-term sedation are tolerance, dependency, and withdrawal. All three are noted to be negative consequences of escalating doses of sedative medications to maintain a desired level of comfort [7].

Tolerance, physical dependency, and withdrawal can occur after the prolonged administration, either intermittent for wound care or continuous for mechanical ventilation, of any agent used for sedation and analgesia [48]. But the exact cellular mechanisms responsible for their development remain poorly defined [17]. All three consequences of sedation require definitive and effective management strategies to treat the problems as well as methods to delay or prevent their occurrence so that these newly recognized issues do not limit the much needed use of sedative and analgesics [48]. Additionally, in the search for the holy grail of ideal sedation, pediatric burn intensivists need to always be wary of the step-brother of sedation which is delirium. For as noted by the RESTORE Investigative team, 54% of ventilator-supported pediatric patients experience a sedation-related adverse event [61].

### Tolerance/dependency

The development of tolerance to continuous infusions of sedatives or analgesia is a well-known complication [49]. Tolerance is defined as a decrease in a drug’s effect or the need to increase the dose to achieve the same effect [48, 62, 63]. The development of tolerance is usually related to changes at or distal to the receptor, generally at a cellular level [48]. The key factors determining tolerance and dependency are occupancy of the receptor by an agonist and the specificity or degree of binding of the agonist at the receptor, but exact cellular mechanisms remain poorly defined [48].

Physiologic dependence is the requirement for continued administration of a sedative or analgesic to prevent signs of withdrawal [62]. Psychological dependence is the need for a substance because of its euphoric effects. Addiction is a complex pattern of behaviors characterized by the repetitive compulsive use of a substance, antisocial or criminal behavior to obtain the drug, and a high incidence of relapse after treatment. Psychological dependency and addiction are extremely rare after the appropriate use of sedative/analgesic agents [48].

Having patients who are both pediatric and burn injured compounds the difficulties of tolerance and dependency. Burn injury causes pathophysiologic alterations to plasma protein concentrations and renal and hepatic function [49]. The skins hold much of the body’s albumin stores, which are directly lost after an extensive burn injury. As a result, plasma free fraction, volume of distribution, and clearance of drugs may be affected. Drugs usually bound by albumin, such as benzodiazepines, may undergo faster clearance in the burn patient, because the drug exists as free fraction for glomerular filtration [49]. Thus, maintaining appropriate sedation and analgesia in pediatric burn patients can be quite challenging and often requires high doses of analgesics and anxiolytics because tolerance quickly develops. Escalating doses of opioids and benzodiazepines provides little additional benefit while increasing the incidence of side effects [52]. Some have postulated that if the sedation regimen is rotated, it may decrease or delay the onset of tolerance, but this theory needs more study prior to widespread adaption [48].

### Withdrawal

Withdrawal, also referred to as abstinence syndrome, includes the physical signs and symptoms that manifest when the administration of a sedative or analgesic agent is abruptly discontinued in a patient who is physically, tolerant [18, 48]. This tends to be the problem of continuous infusions, rather than intermittent dosing for wound care. The risk of withdrawal increases with prolonged administration of high doses of medications, rising to over 50% after 5 days of continuous infusion or around-the-clock administration [21, 62, 64]. Withdrawal is often associated with cumulative doses greater than or equal to 60 mg/kg [49].

The abrupt discontinuation or rapid weaning causes central nervous system hyperirritability, autonomic system dysregulation, gastrointestinal dysfunction, and motor abnormalities [62–65]. Most common symptoms are agitation, irritability, anxiety, insomnia, tachycardia, hypertension, and sweating [62]. But the presenting symptoms vary and may be affected by several factors including the agent involved, the patient’s age, cognitive state, and associated medical conditions [48]. Time to onset of withdrawal symptoms may vary depending on the half-life of the agent and the half-life of active metabolites, which may be several times longer than the parent compound [48, 65]. Withdrawal usually occurs from 1 to 48 h after tapering off or discontinuation of a drug [21], but may be as late as 6 days after commencement of tapering greater than 10% [63]. Thus, signs and symptoms vary from patient to patient in number, severity, and presentation causing confusion and difficulty with diagnosis [48].

Withdrawal estimates vary from 10 - 34% of all PICU patients in Europe to 34–70% in international PICU patients with subsequent increased morbidity, length of stay, and psychological alterations [18, 21]. In pediatric burn centers, 53.7% reported the presence of withdrawal signs and symptoms [31]. The incidence of withdrawal syndrome specific to midazolam has been estimated between 17 and 30% [17].

Withdrawal symptoms for benzodiazepines and opioids had a large overlap for symptoms such as agitation, anxiety, tremors, insomnia, hyperpyrexia, diaphoresis, tachypnea, and tachycardia. Symptoms such as hallucinations, psychomotor agitation with perceptual disorders, depersonalization, and seizures have been described primarily as benzodiazepine withdrawal in PICU patients [17, 48, 49, 62]. Other symptoms include frequent yawning, sneezing, hypertonicity, clonus, and nasal stuffiness [48]. Withdrawal is more likely in patients treated with propofol for >1 day. Symptoms of withdrawal include confusion, tremulousness, hallucinations, seizures, generalized twitching, and jitteriness [48].

To date, there are no reports that demonstrate withdrawal after the prolonged administration of ketamine [48, 65].

### Strategies to mitigate sedation complications

The most common method for decreasing sedative use is the daily scheduled sedation “vacation.” Wake-up protocols, where infusions are held until there is reversal of sedation and patients are able to follow commands, are associated with decreases in mechanical ventilation duration, length of ICU-level care, and complications such as a ventilator-associated pneumonia, deep vein thrombosis, and sepsis [18, 49]. Recent studies have demonstrated successful use of daily interruption in PICUs [18]. Daily sedation interruption in children is feasible and safe [39].

A parallel strategy for decreasing dosing and duration of sedation is to target light sedation levels consistently throughout the day using validated sedation scoring scales. Combining targeted light sedation with daily sedation interruption may be more beneficial than either method alone if sedation doses are reduced and arousal and mobility are facilitated during the ICU stay [34]. Regardless of the strategy employed, a consistent finding across all trials of daily sedation interruption and/or targeted light sedation is that clinical outcomes are improved when sedative doses are significantly reduced [34].

Prevention of withdrawal includes slowly tapering the intravenous administration or, depending on the drug, switching to subcutaneous or oral administration [48]. A validated opioid or benzodiazepine weaning regimen does not exist [49]. Based on a few prospective studies, several authors recommend a daily tapering rate of 1–20% for children who receive benzodiazepines and/or opioids for more than 5–7 days. This strategy did not result in the absence of withdrawal symptoms [62]. Seemingly, much of the burn community agrees, as a survey found that most centers gradually wean off agents to mitigate withdrawal [26]. Adjuncts used to decrease withdrawal include methadone, lorazepam, and clonidine [26].

Unfortunately, again, the PICU practitioners seem to be behind the adult units with incorporating these techniques into daily practice. A Canadian survey of PICUs in 2015 found only 5% of respondents practiced daily interruption of continuous sedation and analgesia [3]. Pediatric burn units appear to be more in line with literature. A survey in 2016 found that 60.9% of pediatric burn units report practicing sedation holidays “always” or “usually” [31].

## Conclusions

Further study needs to be undertaken to create consensus and “gold standards” for pediatric sedation practices. Practices need to be based on valid, high fidelity research. Areas in need of further study include sedation scoring systems, techniques for avoidance of tolerance and dependency, ways to minimize withdrawal and delirium, and mechanisms for limiting sedative medication dosages and duration. Further, we need to further explore non-pharmacologic methods for sedation such as music therapy, maintenance of sleep hygiene, and ideal family presence. As elegantly stated in a survey of pediatric burn centers, best practices for administration and monitoring of pediatric sedation have not been clearly defined, but objective sedative and analgesia measures and targets are worthy pursuits [31].
